# Anti-Oxidative and Anti-Inflammatory Activity of Kenya Grade AA Green Coffee Bean Extracts

**Published:** 2019-11

**Authors:** In-Chul LEE, Jae-Sook LEE, Jeong-Hyun LEE, Yeona KIM, Wi-Young SO

**Affiliations:** 1. Department of Bio-Cosmetic Science, Seowon University, Cheongju, Korea; 2. Department of Beauty Science, Kwangju Women’s University, Kwangju, Korea; 3. Department of Beauty Art, Youngsan University, Busan, Korea; 4. Sports and Health Care Major, College of Humanities and Arts, Korea National University of Transportation, Chungju-si, Korea

**Keywords:** Anti-inflammatory, Anti-oxidative, Cyclooxygenase-2, Green coffee beans, Inducible nitric oxide synthase

## Abstract

**Background:**

Kenya AA green coffee bean extracts were tested for natural ingredients used for anti-oxidative and anti-inflammatory purposes in cosmetic products.

**Methods:**

Anti-oxidative activities were measured by total polyphenol, 1,1-diphenyl-2-picrylhydrazyl (DPPH), and the 2,2′-azinobis (3-ethylbenzothiazoline-6-sulfonic acid) (ABTS) assays. Anti-inflammatory activities were evaluated via nitric oxide (NO) assays, and through quantification of inducible nitric oxide synthase (iNOS), and cyclooxygenase-2 (COX-2) protein expression by western blotting. Data analyses were performed using independent Student’s t-tests, with statistical significance set at P < 0.05.

**Results:**

Total polyphenol content of water and ethanol extract was 169.0 ± 3.1 mg and 300.34 ± 16.6 mg tannic acid/g dry weight, respectively. The DPPH and ABTS radical scavenging activities of all the extracts were significantly increased in a concentration-dependent manner. Kenya AA green coffee bean extracts were toxic at a concentration of 1,000 μg/mL in RAW 264.7 cells. Anti-inflammatory activity as determined by NO assay showed that lipopolysaccharide (LPS)-induced NO was significantly inhibited following treatment with Kenya AA green coffee bean extracts in a concentration-dependent manner. iNOS and COX-2 protein expression was also significantly inhibited following treatment.

**Conclusion:**

These results highlight the potential of Kenya AA green coffee bean extracts as a naturally active anti-inflammatory agent in cosmetic products.

## Introduction

In recent times, along with the improvement in the standard of living and quality of life, there has been an increased social interest in the prevention and treatment of various kinds of inflammation that cause pigmented skin diseases, as well as in reducing the harmful effects induced by environmental factors such as ultraviolet rays and fine dusts. The increased desire for environmentally-friendly materials, has led to a corresponding increase in the number of studies investigating the phytonutrients in natural substances as well as the suitability of the functional materials extracted (1). Inflammatory responses are defense mechanisms that protect against damage from physical stimuli or microbial infections, and often require recovery responses to regenerate the damaged tissue (2). Chronic inflammation causes damage to cellular structures and functions through the transformation of protein, lipids, Deoxyribo-Nucleic Acid (DNA), and Ribo-Nucleic Acid (RNA) by excessive release of active oxygen from inflammatory cells, resulting in oxidative stress (3). Inflammatory mediators such as nitric oxide (NO), tumor necrosis factor-α (TNF-α), interleukin-6 (IL-6), prostaglandin E2 (PGE2), and leukotriene B4 (LTB4) are secreted during the inflammatory response, generating inflammatory factors such as NO and PGE2 by stimulating the expression of genes such as inducible nitric oxide synthase (iNOS) and cyclooxygenase-2 (COX-2) via upregulation of pro-inflammatory cytokine expression, such as TNF-α and IL-6 in the inflammatory response elicited by macrophages. Overproduction of NO can induce angioectasia, tissue damage, mutagenesis, and nerve tissue damage (4–6).

The coffee tree is a dicotyledon that belongs to the family *Rubiaceae*. There are over 100 species of coffee, with the three main types being Arabica, Robusta, and Liberica. The coffee fruit is yellow and red when matured, and the green bean (1.0~1.5 cm in length and 0.6~0.7 cm in width) generally consists of two hemicycles that face each other. The moisture content in coffee seed is approximately 12–13 % and the weight is approximately 0.15–0.5 g (7, 8). This green bean has been reported to contain many polyphenol compounds including, chlorogenic acid which accounts for approximately 12 % of the total content, as well as tannin, lignin, and anthocyanin, in much lower quantities (9–11). Coffee is most commonly known for its application as food product, however, few studies have examined the effect of the green bean are limited.

The objective of this study was to examine the anti-oxidant and anti-inflammatory properties of green coffee beans and their suitability as a novel functional material for us in cosmetic products.

## Methods

### Ingredient and sample extraction

The Kenya green bean used in this study was purchased from Almacielo (Gyeonggi-do, Korea), pulverized and dried for 5 hours at 60 °C. Reflux extraction (Heating Mantle, MS-DM608, MTOP6, Korea) was conducted for 3 hours at 100 °C after added 500 g of distilled water to 50 g of specimen for water extract (WE). Ethanol extract (EE) (70%) was used for stirrer extraction using a heating mantle for 24 hours at room temperature (21–23 °C) after adding 500g of 70% ethanol than 50 g of specimen. Each extract was concentrated using a vacuum evaporator (Rotary Vacuum Evaporator, N-100, EYELA, Tokyo Japan) after which they were vacuum filtered (vacuum pump, DOA-P704-AC, GAST, USA) and freeze-dried. Water content was determined to be 19.12 % and 70% ethanol content was 17.96 %, respectively.

### Reagent and equipment

The 1-1-diphenyl-2-picrylhydrazyl (DPPH) and 2,2'-azino-bis (3-ethylbenzothiazoline-6-sulphonic acid) (ABTS) used for examining anti-oxidant effects, were purchased from Sigma Chemical Co. (St. Louis, MO, USA).

Cell line, Raw 264.7, a macrophage that is used for cell culture and cell toxicity measurement was purchased from Korea cell line bank (Korea Cell Line Bank, Seoul, Korea), and Dulbecco’s modified Eagle medium (DMEM), fetal bovine serum (FBS), phosphate buffered saline (PBS), penicillin/streptomycin, trypsin for cell culture was purchased from Thermo Scientific HyClone (Logan, UT, USA). Haemacytometer (Marienfeld, Germany) and 3-(4,5-dimethyl-thiazol-2-yl)-2,5-diphenyl-tetrazoliumbromide (MTT), which were used for cell toxicity measurement were purchased from Sigma Chemical Co. (St. Louis, MO, USA), and dimethyl sulfoxide (DMSO) was purchased from Bioshop (Burlington, ON, Canada). Lipopolysaccharide (LPS) and griess regent used for anti-inflammation measurement were purchased from Sigma Chemical Co. (St. Louis, MO, USA). Furthermore, primary antibody iNOS, COX-2 and secondary antibody anti-mouse for protein expression measurement were purchased from Santa Cruz (CA, USA).

The equipment used in the study were vacuum evaporator (Rotary Vacuum Evaporator, N-100, EYELA, Tokyo Japan), Digital Precise Shaking Water Bath (WSB-45,Wisd, Korea), ELISA reader (Tecan, Austria), Image Quant LAS 4,000 (GE Healthcare Bio-Sciences AB, Uppsala, Sweden), CO_2_ incubator (vision scientific, Korea), pH meter (Mettler-Toledo AG, Swltzerland), centrifuge (Hanil Science Industrial Co. Korea), autoclave (JS Research Inc, Korea), and Davinch-Chemi™ imager CAS-400SM System (Davinch-K Co, Korea).

### Total phenolic contents

Total polyphenol content was measured by modifying the Folin-Denis method (12). The extract was diluted with 2 mL of distilled water, 2 mL of which was mixed with an additional 2 mL of 50 % Folin-Ciocalteu reagent (Sigma Chemical Co., St. Louis, MO, USA) and incubated at room temperature (21–23 °C) for 3 minutes. Then, 2 mL of 10 % CaCO_3_ was added and mixed, and incubated at room temperature (21–23 °C) for 1 hour. Optical density at 700 nm was measured (ELISA reader, Tecan, Austria).

### Measurement of electron donating abilities (EDA)

Measurement of EDA was conducted, using a modified Blois’s method (13). DPPH solution (0.2 mM; 60 μL) (Sigma Chemical Co., St. Louis, MO, USA) was dissolved in 100% ethanol and samples from each concentration (120 μL) were added to the microwell plate, and allowed to react for 15 minutes at room temperature (21–23 °C), at which point optical density at 517 nm was measured by using microplate reader. EDA were expressed as optical density reduction ratio of specimen added and non-specimen added groups. The DPPH (60 μL) and 120 μL of sample solution of 5, 10, 50, 100, 500, 1,000 μg/mL concentration were mixed and left at room temperature (21–23 °C) for 15 minutes; the optical density was then measured by using microplate reader at 517 nm. The EDA were expressed as optical density reduction ratio of specimen added group and non-specimen adding group.

$$\text{EDA}\;(\%)=(1-\frac{\text{Optical}\;\text{density}\;\text{of}\;\text{specimen}\;\text{added}\;\text{group}}{\text{Optical}\;\text{density}\;\text{of}\;\text{non-added}\;\text{group}})\times 100$$

### ABTS+ cation radical scavenging activity assay measurement

ABTS radical scavenging ability was measured by Nicoletta’s method (14). Briefly, ABTS (7 mM; 5 mL) (Sigma Chemical Co., St. Louis, MO, USA) and K_2_S_2_O_8_ (140 mM; 88 μL) (Kanto Chemical Co., INC., Kanto, Japan) were combined and allowed to react for 14–16 hours in the dark. This solution was then mixed with absolute 100% ethanol at a ratio of 1:88. ABTS solution, which had been adjusted to an optical density of 0.700 ± 0.002 at 734 nm was used. Sample solution (150 μL) and ABTS solution (3 mL) were mixed in a vortex for 30 seconds, reacted at room temperature (21–23 °C) for 2.3 minutes, and the optical density was then measured at 734 nm.

$$\text{ABTS}\;\text{scavenging}\;\text{activity}\;(\%)=(1-\frac{\text{Optical}\;\text{density}\;\text{of}\;\text{specimen}\;\text{added}\;\text{group}}{\text{Optical}\;\text{density}\;\text{of}\;\text{non-added}\;\text{group}})\times 100$$

### Cell culture

Dulbecco's Modified Eagle’s Medium (DMEM) (Thermo Scientific HyClone, Logan, UT, USA), supplemented with 10 % fetal bovine serum (FBS) (Thermo Scientific HyClone, Logan, UT, USA) and 1 % penicillin/streptomycin (100 U/mL) (Thermo Scientific HyClone, Logan, UT, USA) was used for cell cultures, and was subcultured by adapting it to a 5 % CO_2_ incubator at 37 °C.

### Measurement of cell survival rate by MTT assay

Cell survival rate was measured by Carmichael’s method (15). Briefly, RAW 264.7 cells (macrophage cell line) (Korea Cell Line Bank, Seoul, Korea) were divided into 0.18 mL for a final concentration of 1×10^5^ cells/well in 96 well plates. A sample (200 μL) of each concentration was added to the cells and cultured in a 5%, CO_2_ incubator for 24 hours at 37 °C. The control group was cultured in the same conditions by adding the same amount of distilled water as specimen. Two-hundred microliters of MTT solution (5 mg/mL) (Sigma Chemical Co., St. Louis, MO, USA) was added and cultured for 4 hours, after which the culture media was removed, and 150 μL of DMSO (Bioshop, Burlington, ON, Canada) was added to each well and incubated for 30 minutes at room temperature (21–23 °C). Finally, the optical density was measured, using an ELISA reader (Tecan, Austria) at 540 nm. The cell survival rate was expressed as optical density reduction ratio of specimen added group and non-specimen adding group.

$$\text{Cell}\;\text{Survival}\;\text{rate}\;(\%)=(1-\frac{\text{Optical}\;\text{density}\;\text{of}\;\text{specimen}\;\text{added}\;\text{group}}{\text{Optical}\;\text{density}\;\text{of}\;\text{non-added}\;\text{group}})\times 100$$

### NO inhibition activation measurement

The content of NO produced from RAW 264.7 cells was measured in the form of NO_2_ detectable in the culture supernatant, using the method established by Green et al (16) with griess reagent (Sigma Chemical Co., St. Louis, MO, USA). RAW 264.7 cells were seeded (5×10^5^ cells/well) in 6-well plates and washed twice with 1× PBS (Thermo Scientific HyClone, Logan, UT, USA) after culturing in the CO_2_ incubator at 37 °C for 24 hours. LPS (1 μL/mL) (Sigma Chemical Co., St. Louis, MO, USA) was treated with a sample solution of each concentration 2 hours after removal, cultured for 24 hours to obtain the supernatant 100 μL, reacted for 10 minutes in a 96-well plate by adding the same amount of griess reagent, and the optical density was measured at 540 nm. The level of NO inhibition activation was expressed as optical density reduction ratio of specimen added group and non-specimen adding group.

$$\text{NO}\;\text{inhibition}\;\text{activity}\;(\%)=(1\frac{\text{Optical}\;\text{density}\;\text{of}\;\text{specimen}\;\text{added}\;\text{group}}{\text{Optical}\;\text{density}\;\text{of}\;\text{non-added}\;\text{group}})\times 100$$

### Measurement of protein expression through western blot analysis

The cell line, RAW 264.7, was seeded at 1×10^6^ cells/well in a 100 mm tissue culture dish and cultured for 24 hours to acclimatize the cells to identify basal levels of iNOS and COX-2 activation. The media was then removed and LPS at a concentration of 1 μL/mL was added to the cells for 2 hours. The cells were then incubated for 24–48 hours with media that was treated with green coffee extract of 5, 10, 50, 100 μg/mL concentration. The media was removed and the cells were washed twice with phosphate buffered saline (PBS). One-hundred microliters of complete mini 1 tab (Thermo Scientific HyClone, Logan, UT, USA) dissolved into 10 mL of radio-immunoprecipitation assay (RIPA) buffer (Sigma Chemical Co., St. Louis, MO, USA) and added to the cells. Cell solutions were centrifuged for 20 minutes at 13,200 rpm and 4 °C. The total protein concentration in the supernatants was quantified using the bicinchoninic acid (BCA) assay kit. Proteins (20 μL) were separated via 10 % SDS-PAGE. The separated proteins were treated with blocking buffer (5 % skim milk in tris-buffered saline with Tween20; TBST) for 1 hour and later transferred to polyvinylidene fluoride (PVDF) membranes, using a transfer device (BIORAD). The primary antibodies specific for iNOS (C-11), COX-2 (H-3), and β-actin (C-4) (Santa Cruz, CA, USA) were diluted and applied to the membranes overnight at 4 °C, and then washed thrice with TBST for 10 minute intervals. Anti-rabbit (sc-2491) iNOS and COX-2 secondary antibodies (Santa Cruz, CA, USA) were then applied together with anti-mouse (sc-516102) β-actin secondary antibody (Santa Cruz, CA, USA), diluted at 1:1,000, incubated for 2 hours at room temperature (21–23 °C). Band visualization and quantification were conducted using LAS 4,000 (Davinch-K Co, Korea) after washing with TBST 3 times.

### Statistical Analysis

All results were presented as mean ± standard deviation (SD). Data analyses were performed using independent Student’s *t*-tests and one-way analysis of variance (ANOVA) to verify the intergroup differences. Tukey (Post-hoc testing) was conducted to specifically confirm which groups showed significant differences. The analyses were performed using SPSS version 21.0 (IBM Corp., Armonk, NY, USA) and statistical significance was set at *P<* 0.05.

## Results

The results obtained for the total polyphenol content in green bean extract using tannic acid as standard material are provided in Table 1. Hot water extract showed 169.0 mg tannic acid/g content and 70% ethanol extract showed 300.34 mg tannic acid/g content resulting in 1.8 times higher content in ethanol extract than in hot water extract.

**Table 1:** Total phenol content in Kenya grade AA green coffee bean extracts

***Sample***	***Total polyphenols***
Water extract (mg/g dry)	169.00 ± 3.06
Ethanol extract (mg/g dry)	300.34 ± 16.62

Total polyphenol content of water and ethanol extract was determined to be 169.0 ± 3.1 mg and 300.34 ± 16.6 mg tannic acid/g dry weight, respectively. The results of the DPPH and ABTS radical scavenging activity assays for all extracts significantly increased in a concentration-dependent manner. Kenya AA green coffee bean extracts were found to be toxic at a concentration of 1,000 μg/mL in RAW 264.7 cells. Anti-inflammatory activity as determined by the NO assay revealed that LPS-induced NO was significantly inhibited following treatment with Kenya AA green coffee bean extracts and decreased in a concentration-dependent manner. Further, the expression of iNOS and COX-2 proteins was significantly inhibited following treatment.

## Discussion

### Total polyphenol content

Many anti-oxidants, vitamins, and anti-oxidant-activating phytochemicals are contained in plants. Polyphenols are secondary metabolites and aromatic compounds that contain more than 2 phenolic hydroxyl (-OH) groups in one molecule. Polyphenolic compounds in plants are known to exhibit several activities such as anti-oxidant activity (17, 18).

### Measurement of EDA

DPPH radical scavenging activity is a method of measuring anti-oxidation ability by using an index that shows the degree of bleaching of violet from reduction by aromatic compounds and aromatic amines due to an antioxidant’s electron donating ability (19).

**Fig. 1:**
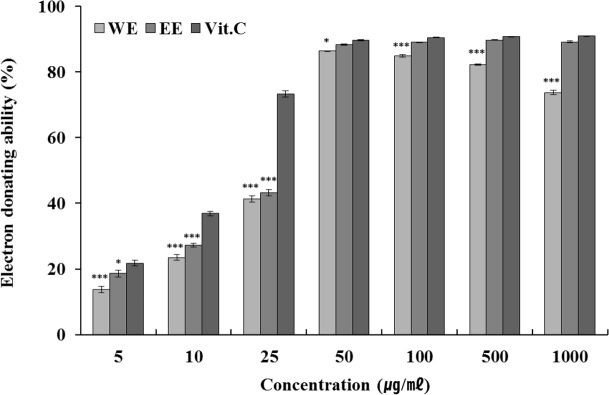
Electron donating abilities of Kenya AA green coffee bean extracts Each value represents mean ± standard deviation of three individual experiments. Tested by one-way analysis of variance, *P < 0.05, *** *P* < 0.001 compared with Vit. C: Ascorbic acid; Tukey’s post-hoc testing was performed. 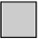
WE: Kenya AA green coffee beans extracted with water (n=3) 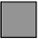
EE: Kenya AA green coffee beans extracted with ethanol (n=3) 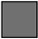
Vit.C: Ascorbic acid (n=3)

The result of the measurement of electron donating ability of green bean hot water and ethanol extracts is shown in Fig. 1. Hot water extract exhibited the highest effect (83 %) at a concatenation of 50 μg/mL, proving that it is effective at relatively low concentration. Moreover, the effect of ethanol extract increased with increasing concentration, and the highest effect (89.14 %) was observed at the highest concentration of 1,000 μg/mL.

### ABTS radical scavenging activity measurement

**Fig. 2:**
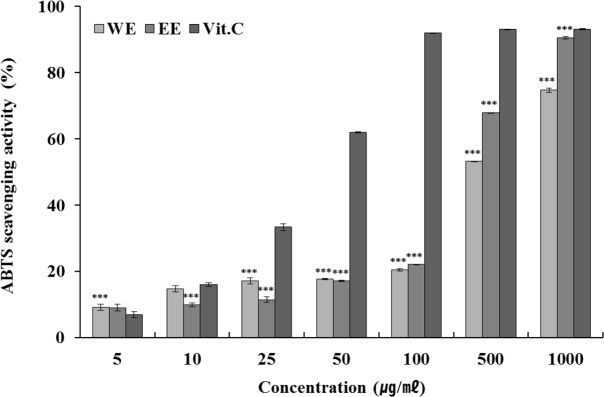
ABTS radical scavenging ability of Kenya AA green coffee bean extracts Each value represents mean ± standard deviation of three individual experiments. Tested by one-way analysis of variance, * *P* < 0.05, *** *P* < 0.001 compared with Vit. C: Ascorbic acid; Tukey’s post-hoc testing was performed. 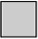
WE: Kenya AA green coffee beans extracted with water (n=3) 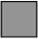
EE: Kenya AA green coffee beans extracted with ethanol (n=3) 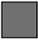
Vit.C: Ascorbic acid (n=3)

Measuring the antioxidant capacity of ABTS radicals is a method that involves the use of the bleaching reaction principle, which is based on the fact that activated ABTS radical is bleached into an antioxidant with a unique blue-green color. Furthermore, ABTS radical scavenging activity exhibits a relatively positive correlation with the concentration of phenolic content (20, 21). The result of the measurement of ABTS radical scavenging activity of green bean hot water and ethanol extract is shown in Fig. 2. The effect of ABTS radical scavenging activity was seen to increase in a density-dependent way in both green bean hot water and in the ethanol extract. Hot water and ethanol extract showed a 74.75 % and 90.47 % effect, respectively following treatment with 1,000 μg/mL. These effects were significantly higher than that observed for Vitamin C, suggesting the superiority of green bean hot water and ethanol extract anti-oxidant agents.

### Measurement of cell survival rate by MTT assay

The MTT assay, which was conducted to identify the rate of cell survival, uses a citrine color substrate and a method of examination that measures the amount of formazan in living cells by measuring the optical density of dark red formazan. The reaction does not occur in dead cells as formazan is produced by reduction from respiratory chain enzyme within the mitochondria of living cells (22).

**Fig. 3:**
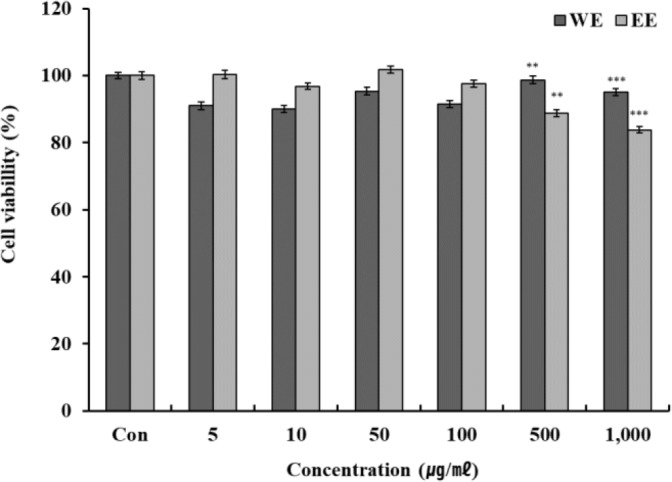
Cell viability of Kenya AA green coffee bean extracts on macrophage cells (RAW 264.7) RAW 264.7 cells were incubated for 24 hours in DMEM containing 10 % FBS and treated with increasing concentrations of Kenya AA green coffee bean extracts for 24 hours and cell viability was measured by MTT assays. Each value represents mean ± standard deviation of three individual experiments. ** *P* < 0.01, *** *P* < 0.001, tested by independent Student’s *t*-tests. 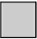
WE: Kenya AA green coffee beans extracted with water (n=3) 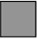
EE: Kenya AA green coffee beans extracted with ethanol (n=3)

The results from the MTT assay cell survival rate analysis in RAW 264.7 cells (macrophage cell line) treated with green bean hot water and ethanol extract are presented in Fig. 3. Green bean hot water and ethanol extract showed > 80 % cell survival at all concentrations; therefore, the NO and western blot assays were conducted using 100 μg/mL, which showed more than 90 % cell survival rate.

### Measurement of nitric oxide (NO) inhibition activation

NO is a vitreous molecule that is produced from L-arginine by NO synthetase; it is involved in various biological processes including, immune reactions, cell toxicity, nigrostriatal system, vaso-relaxation. It also has a concentration-dependent effect on the maintenance of cell function and is capable of inducing cell toxicity (23).

**Fig. 4:**
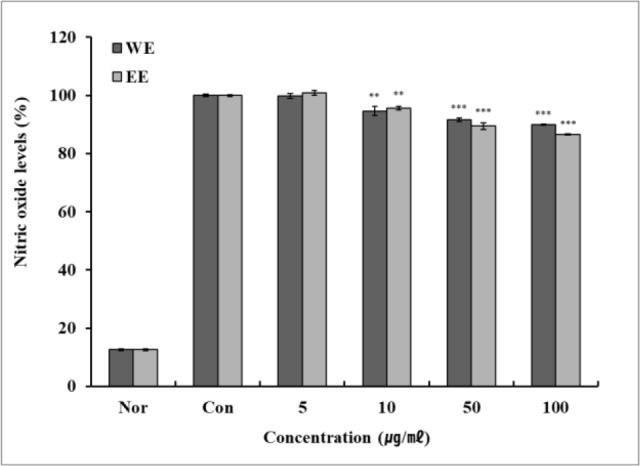
Effect of Kenya AA green coffee bean extracts on production of nitric oxide in RAW 264.7 cell Effect of Kenya AA green coffee bean extracts on NO production in LPS-induced RAW 264.7 cells. RAW 264.7 cells (5×10^5^ cells) were treated with Kenya AA green coffee beans and LPS (1 μg/mL) for 24 hours. Each value represents mean ± standard deviation of three individual experiments. ** *P* < 0.01, *** *P* < 0.001, tested by independent Student’s *t*-tests 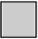
WE: Kenya AA green coffee beans extracted with water (n=3) 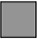
EE: Kenya AA green coffee beans extracted with ethanol (n=3)

The effect of green bean hot water and ethanol extract on the production of NO, which is a reactive oxygen species and is known to play an important role in the induction of inflammation, was evaluated. Fig. 4, shows that the LPS-treated group showed higher NO expression compared to the LPS-non treated group did. Moreover, green bean hot water and ethanol extract-treated groups reduced NO expression compared to that of the LPS-treated group. It was also found that both extracts exhibited inhibitory effects on inflammatory processes that nitric oxide is a factor that induces inflammation. Reduction of NO production in macrophages can be considered as a process to inhibit inflammation in RAW 264.7 cells.

### Measurement of inhibition effect on iNOS and COX-2 protein expression

Inflammation mediators such as nitric oxide (NO), prostaglandin E_2_ (PGE_2_), and inflammatory cytokines are secreted during the inflammatory process. Of these mediators, NO is synthesized by NO synthase (NOS) in L-arginine, and under pathological conditions, iNOS plays important roles in NO production. Cyclooxygenase (COX), another inflammatory factor, transfers arachidonic acid to prostaglandins. There are two types of COX, namely, COX-1 and COX-2. COX-2 is expressed during an inflammatory response and PGE_2,_ which is produced by COX-2 is an inflammatory mediator, involved in pain, pyraxy, etc., and has been shown to be associated with promotion of neovascularization by influencing inflammation and immune responses (6, 24).

**Fig. 5:**
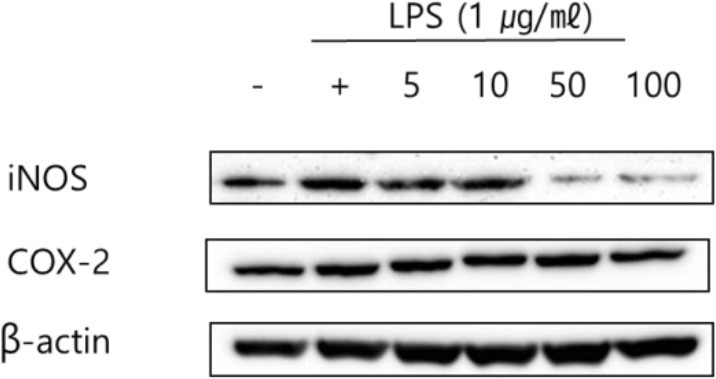
iNOS and COX-2 protein expression rate of Kenya AA green coffee beans extracted with water on RAW 264.7 cell. After RAW 264.7 cells (1×10^6^ cells) were cultured in serum-free media for 1 hour, the cells were treated with 5, 10, 50, and 100 μg/mL of Kenya AA green coffee beans extracted with water for 24 hours

**Fig. 6:**
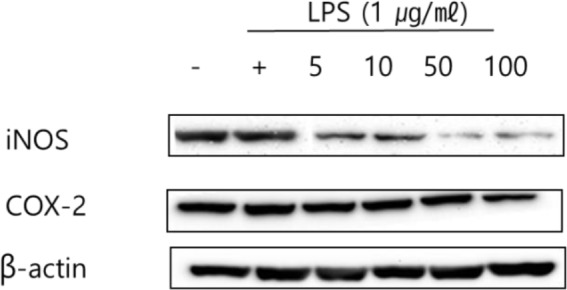
iNOS and COX-2 protein expression rate of Kenya AA green coffee beans extracted with ethanol on RAW 264.7 cell. After RAW 264.7 cells (1×10^6^ cells) were cultured in serum-free media for 1 hour, the cells were treated with 5, 10, 50, and 100 μg/mL of Kenya AA green coffee beans extracted with ethanol for 24 hours

Western blot analysis was conducted in this study to measure the inhibitory effect of iNOS and COX-2 on the expression of inflammatory mediators. The results obtained showing the effect of treating RAW 264.7 cells with 5, 10, 50 and 100 μL/mL of green bean hot water and ethanol extract are presented in Fig. 5 and 6. β-actin, which was the housekeeping gene (positive control) showed no difference in expression in any cell type or culture condition. Fig. 3 shows that the expression of iNOS and COX-2, which were increased by LPS in the cell, were inhibited in a concentration-dependent manner in RAW 264.7 cells treated with green bean hot water and ethanol extract.

It was also observed that green bean hot water and ethanol extract exhibited superior inhibitory effects on iNOS and COX-2, illustrating its potential for use as an anti-inflammatory agent.

## Conclusion

These results highlight the potential of Kenya AA green coffee bean extracts to be used as a naturally active and anti-inflammatory agent in cosmetic products.

## Ethical considerations

Ethical issues (Including plagiarism, informed consent, misconduct, data fabrication and/or falsification, double publication and/or submission, redundancy, etc.) have been completely observed by the authors.

## References

[1] Jin KS Oh YN Park JA (2012). Anti-oxidant, anti-melanogenic, and anti-inflammatory activities of *Zanthoxylum schinifolium* extract and its solvent fractions. Korea J Microbiol Bio, 40( 4): 317–379.

[2] Lawrence T Willoughby DA Gilroy DW (2002). Anti-inflammatory lipid mediators and insights into the resolution of inflammation. Nat Rev Immunol, 2( 10): 787–795. 12360216 10.1038/nri915

[3] Ryter SW Kim HP Hoetzel A (2007). Mechanisms of cell death in oxidative stress. Antioxid Redox Signal, 9( 1): 49–89. 17115887 10.1089/ars.2007.9.49

[4] Shin JS Kim JM An WG (2012). Anti-inflammatory effect of red ginseng through regulation of MAPK in lipopolysaccharide-stimulated RAW 264.7. J Physiol Pathol Korean Med, 26( 1): 293–300.

[5] Knowles RG Moncada S (1992). Nitric oxide as a signal in blood vessels. Trends Biochem Sci, 17( 10): 399–402. 1280869 10.1016/0968-0004(92)90008-w

[6] Nathan C (1992). Nitric oxide as a secretory product of mammalian cells. FASEB J. 6( 12): 3051–3064. 1381691

[7] Seo HS (2006). Development of sensory and sensibility evaluations of coffee and analysis of coffee preference types with segmented coffee consumers. Seoul National University . Seoul, Korea .

[8] Yoo DJ (2013). Coffee inside ( 5th edition ). Lion company . Seoul, Korea .

[9] Lopez-Garcia E van Dam RM Willett WC (2006). Coffee consumption and coronary heart disease in men and women: A prospective cohort study. Circulation, 113( 17): 2045–2053. 16636169 10.1161/CIRCULATIONAHA.105.598664

[10] Farah A Donangelo CM (2006). Phenolic compounds in coffee. Braz J Plant Physiol, 18( 1): 23–36.

[11] Esquivel P Jimenez VM (2012). Functional properties of coffee and coffee by-products. Food Res Int, 46( 2): 488–495.

[12] Singleton VL Rossi JA (1965). Colorimetry of total phenolics with phosphomolybdic: phosphotungstic acid reagents. Am J Enol Viticult, 16: 144–158.

[13] Blois MS (1958). Antioxidant determinations by the use of a stable free radical. Nature. 181: 1199–1120.

[14] Nicoletta F Roberta K Min Y (1999). Screening of dietary carotenoids and carotenoid-rich fruit extracts for antioxidant activities applying 2, 2'-azinobis (3-ethylbenzothiazoline-6-sulfonic acid) radical cation decolorization assay. Methods Enzymol, 299: 379–389.

[15] Carmichael J DeGraff WG Gazdar AF (1987). Evaluation of a tetrazolium-based semiautomated colorimetric assay: assessment of chemosensitivity testing. Cancer Res, 47( 4): 936–942. 3802100

[16] Green LC Wagner DA Glogowski J (1982). Analysis of nitrate, nitrite, and [15N] nitrate in biological fluids. Anal Biochem, 126( 1): 131–138. 7181105 10.1016/0003-2697(82)90118-x

[17] Choi SY Lin SH Ha TY (2005). Evaluation of the estrogenic and antioxidant activity of some edible and medical plant. Korean J Food Sci Technol, 37( 4): 549–556.

[18] Lee JH Lee SR (1994). Analysis of phenolic substances content in Korean plant food. Chem Pham Bull, 26: 310–316.

[19] Choi CS Song ES Kim JS (2003). Antioxidative Activities of Castanea Crenata Flos. Methanol Extracts. Korean J Food Sci Technol, 35: 1216–1220.

[20] Arnao MB (2000). Some methodological problems in the determination of antioxidant activity using chromogen radicals: a practical case. Trend Food Sci Technol, 11( 11): 419–421.

[21] Jeong JA Kwon SH Lee CH (2007). Screening for anti-oxidative activities of extracts from aerial and underground parts of some edible and medicinal ferns. Korean J Plant Resour, 20: 185–192.

[22] Ukeda H Maeda S Ishii T (1997). Spectrophotometric assay for superoxide dismutase based on tetrazolium salt 3’-1-(phenylamino)-carbonyl-3,4-tetrazolium]- bis(4-methoxy-6-nitro) benzenesulfonic acid hydrate reduction by xanthine-xanthine oxidase. Anal Biochem, 251( 2): 206–209. 9299017 10.1006/abio.1997.2273

[23] Kim JY Jung KS Jeong HG (2004). Suppressive effects of the kahweol and cafestol on cyclooxygenase-2 expression in macrophages. FEBS Lett, 569 ( 1–3 ): 321 – 326 . 15225655 10.1016/j.febslet.2004.05.070

[24] Masferrer JL Zweifel BS Manning PT (1994). Selective inhibition of inducible cyclooxygenase 2 in vivo is antiinflammatory and nonulcerogenic. Proc Natl Acad Sci, 91( 8): 3228–3232. 8159730 10.1073/pnas.91.8.3228 PMC43549

